# Lipsmacking Imitation Skill in Newborn Macaques Is Predictive of Social Partner Discrimination

**DOI:** 10.1371/journal.pone.0082921

**Published:** 2013-12-18

**Authors:** Elizabeth A. Simpson, Annika Paukner, Valentina Sclafani, Stephen J. Suomi, Pier F. Ferrari

**Affiliations:** 1 Eunice Kennedy Shriver National Institute of Child Health and Human Development, Laboratory of Comparative Ethology, Poolesville, Maryland, United States of America; 2 Dipartimento di Neuroscienze, Università di Parma, Parma, Italy; University of Milan, Italy

## Abstract

Newborn rhesus macaques imitate facial gestures even after a delay, revealing the flexible nature of their early communicative exchanges. In the present study we examined whether newborn macaques are also sensitive to the identities of the social partners with whom they are interacting. We measured infant monkeys' (*n* = 90) lipsmacking and tongue protrusion gestures in a face-to-face interaction task with a human experimenter in the first week of life. After a one-minute delay, the same person who previously presented gestures or a different person returned and presented a still face to infants. We had two primary predictions: (1) infants would demonstrate higher rates of overall gesturing, and especially lipsmacking—an affiliative gesture—to a familiar person, compared to a novel person, and (2) infants' imitative skills would positively correlate with gestures to familiar, but not unfamiliar, social partners, as both abilities may reflect a strong general social interest. We found that overall infants did not produce more gestures or more lipsmacking when approached by a familiar person compared to a novel person; however, we did find individual differences in infants' social responsiveness: lipsmacking imitation was positively correlated with lipsmacking during the return period when the person was the same (*p* = .025), but not when the person was novel (*p* = .44). These findings are consistent with the notion that imitative skill is reflective of infants' more general interest in social interactions.

## Introduction

Neonatal imitation is similar in human and macaque infants [1], [2] and appears to be underpinned by the mirror neuron system, which is likely functioning from birth [3]. Newborn macaques remember lipsmacking gestures after a delay and initiate social interactions using the same gesture, revealing that neonatal imitation is not a simple reflex or innate preset motor program, but rather a sophisticated and flexible form of communication [4]. Researchers have proposed at least two non-exclusive functions of neonatal imitation. One function is that neonatal imitation maintains social interactions and bonding between infants and caregivers [5], [6]. By imitating the caregiver newborns communicate their propensity to be emotionally engaged and their interest in social interactions, which promotes social communicative exchanges and strengthens the relationship. A second proposal suggests that neonatal imitation serves a cognitive function, whereby infants can increase their knowledge of the world, including their understanding of actions, people, and social communication norms [7]–[9]. According to this conception of imitation, infants employ imitation as a tool through which they can attempt to understand puzzling events (e.g., modeled actions [9]) and unknown individuals (e.g., social partners [8], [10]). In this way, neonatal imitation may serve an identity function, allowing infants to identify and communicate with social partners when they re-encounter them [8], [10].

According to the identity function hypothesis, infants need to remember both the actions and the person who made those actions so they can produce a matching “probe” action, which serves as a test of whether the present person is the same person as before. Meltzoff and Moore [10] examined this question with 6-week-old human infants viewing two gestures—tongue protrusion (TP) and mouth opening (MO)—performed by two models. Infants first viewed the first gesture performed by one person for 90 sec, then that person departed and a different person returned and produced a different gesture for 90 sec. Infants who visually tracked social partners' entrances and exits—and therefore, were more informed that the person may have changed—were more likely to imitate the gestures of a new person, whereas infants who did not visually track the social partners were more likely to produce the gestures of the first person, perhaps because they did not realize there was a change in their social partner. Thus, one way to test whether imitation serves a cognitive function (namely, social partner identity discrimination) is to measure infants' tracking of social partners to see if it predicts their rate of producing the previously viewed gesture.

In the present study, we had two primary goals. We examined (1a) whether macaque newborns, like humans, discriminate social partners, (1b) whether infants' visual tracking of social partners aids in this discrimination, and (2) whether infants' responsivity to novel and familiar social partners is related to infants' imitative performance. We measured infants' production of two gestures—lipsmacking (LPS) and tongue protrusion (TP)—in a face-to-face interaction task with a human experimenter when infants were 1–8 days old. After gesture presentation, the human model exited infants' visual field, and after a delay period of one minute, the same human model or a different human model approached the infants and presented a still face. The model exhibited a still face during the return period, rather than a novel gesture, as previous work has used [10], because we were interested in examining infants' initiation of the social interaction and whether they would use the previously seen gesture to probe the returning model. We measured infants' LPS and TP responses during this last return period and hypothesized that if infants can discriminate social partners they will be more likely to initiate an interaction with a familiar social partner (with whom they have previously interacted) than a novel social partner, and they will either use the gestures they saw that individual present previously or will respond with increased LPS specifically (an affiliative gesture). We also tested whether macaque infants, like humans, track social partners and whether tracking aids in their person discrimination. Specifically, we predicted that macaque infants, like humans [10], would produce previously seen gestures when confronted with an unfamiliar person during the return period, but only in sessions in which infants failed to visually track social partners' exits; in contrast, in sessions in which infants tracked the social partners' exits, infants would be less likely to produce the previously seen gesture upon being confronted with an unfamiliar person in the return period. Finally, we examined whether individual differences in imitative capacity are associated with individual differences in social partner discrimination. If imitation and social partner recognition both are reflective of general social interest, then we predict that when the return person is the same, there will be a positive association between facial gesture imitation and response to a still face with gestures made previously by that model. In contrast, when the return person is novel, we predicted a negative association between imitative skill and the rates of gestures (matching those previously performed by a different model) during a still face return period.

## Methods

### Subjects

We tested 90 infant rhesus macaque monkeys (*Macaca mulatta*), 40 females and 50 males. Four infants were born via C-section due to labor complications; all others were born without intervention. Infants appeared to be at term and birth weights were normal. Infants were separated from their mothers on day 1 post-partum for unrelated and ongoing experiments and were subsequently raised in a neonatal primate nursery. There, infants were individually housed in incubators (51 cm×63.8 cm×64.3 cm) containing an inanimate surrogate mother covered with fleece fabric and various age-appropriate environmental enrichment (e.g., loose pieces of fleece fabric, soft plush animals, plastic toys). Infants could see and hear other infants but could not touch them. The incubator was maintained at a temperature of ∼27°C and at 50%–55% humidity. Lights were on from 07:00 to 21:00. All animals were fed Similac formula (Ross Laboratories, Columbus, Ohio, United States). Infants were hand-fed until they were old enough to feed independently, usually by day 4. Once the animals reached 1 month of age, Purina High Protein Monkey Chow (#5038) (Purina, St. Louis, Missouri, United States) and water were available ad libitum. For further details on rearing practices, see [11].

This study was carried out in accordance with the recommendations in the Guide for the Care and Use of Laboratory Animals. The *Eunice Kennedy Shriver* National Institute of Child Health and Human Development's Animal Care and Use Committee approved this study (protocol: 11-043).

### Materials and Procedure

**Figure 1:**
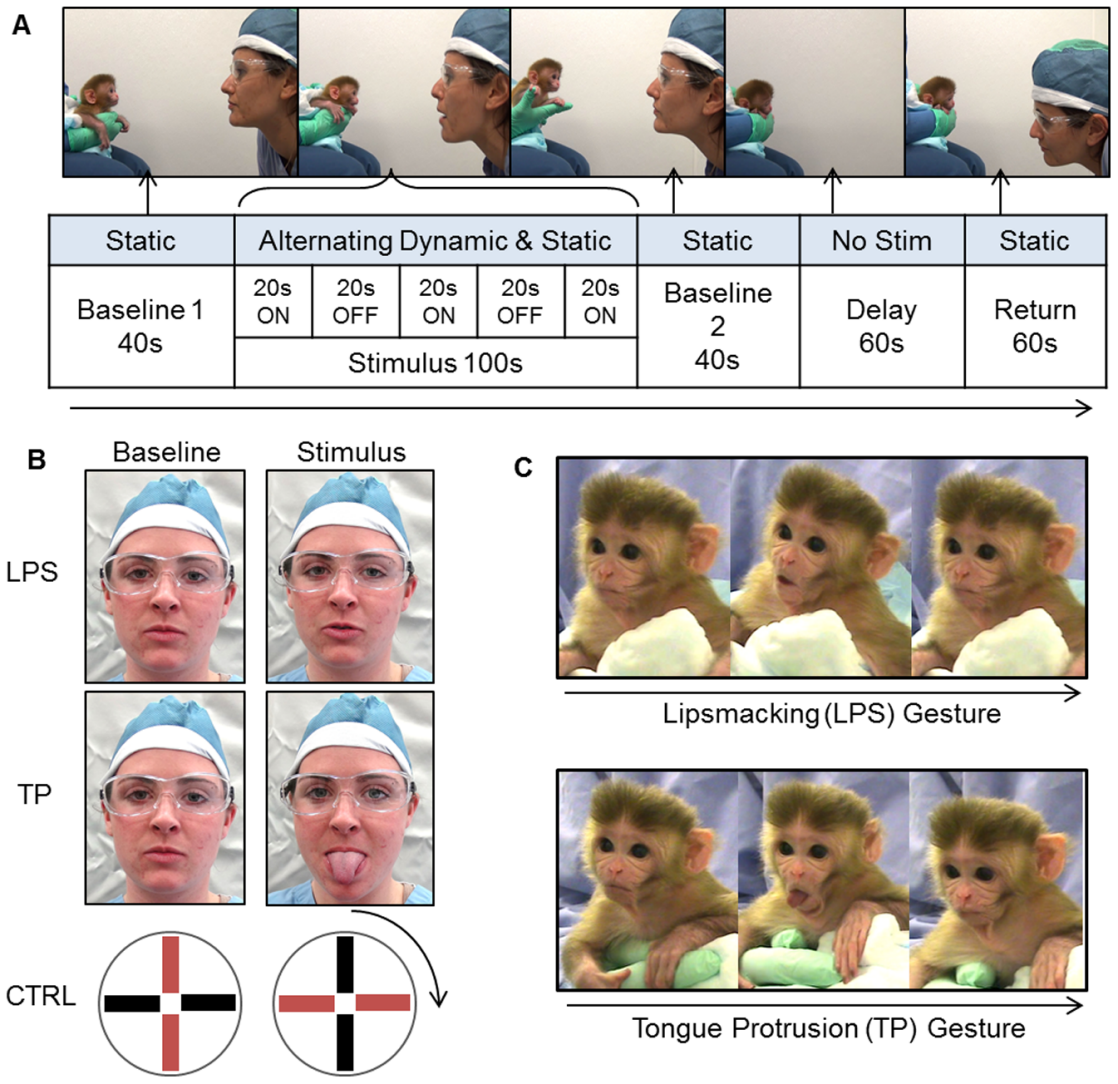
Methodological details. Procedure (A), examples of lipsmacking, tongue protrusion, and control stimuli (B), and examples of infants' gestures (C). Human models portrayed here have given written informed consent, as outlined in the PLOS consent form, for publication of their photographs.

Infants were tested three times a day, every other day, in the first week of life (days 1 or 2, 3 or 4, 5 or 6, and 7 or 8). Testing took place at ∼9:30am, ∼12:30pm, and ∼2:30pm; there was at least an hour between test sessions. The procedure was as detailed by Paukner et al. [4]. Infants were only tested if they were in an awake, attentive state. A demonstrator silently presented infants with three stimuli, once per day (one stimulus during each of the three sessions), at a distance of approximately 30 cm: lipsmacking gestures (LPS, rapid opening and closing of the mouth; ∼100 openings/20 s), tongue protrusion gestures (TP, protrusion and retraction of the tongue; ∼seven openings/20 s), and a nonsocial control condition (CTRL; a white plastic disk with black/red or green/yellow orthogonal stripes, slowly rotated 180° clockwise and counter-clockwise), Figure 1b. The purpose of the CTRL condition was to assess the effect of a non-biological stimulus and movement, similar in size to the human face, on infant macaque behavior.

In each test session, one experimenter held the infant, a second experimenter—the demonstrator—served as the source of the stimuli, and a third experimenter was the time-keeper who ensured stimuli were presented for appropriate lengths. At the beginning of a trial, there was a 40 sec baseline (BASELINE 1), in which the demonstrator displayed a calm, neutral facial expression (or the still disk in CTRL), Figure 1A. The demonstrator then gestured (LPS or TP, or rotating the disk in CTRL) for 20 sec, followed by a still, neutral facial expression (still disk in CTRL) period for 20 sec. This gesture-still face sequence was repeated, for a total of three 20-sec dynamic periods separated by two 20-sec static periods (STIMULUS). This was followed by a final still face expression period lasting 40 sec (BASELINE 2). The demonstrator then walked behind the infant, out of view. The first experimenter continued to hold infants without any particular visual focus (no stimuli were presented), for 60 sec (DELAY). We used a delay of one minute because newborn macaques remember gestures after a delay of this length [4], a delay of this length is naturalistic for mother-infant pairs [12], and in primates memory after a one-minute delay is considered to be episodic-like [13]. After this delay period the demonstrator/disk—either the original demonstrator/disk or a new demonstrator/disk—returned to the initial position in front of the infant and displayed a still face, neutral expression (still disk in CTRL) for 60 sec (RETURN). In total, the test was 5 minutes.

The return person and disk during the return period were the same for some infants (*n* = 54; these data are a subset of those previously published in [12]) and were different for others (*n* = 36). In both cases, testing conditions were identical, including the testing environment and stimuli; the only variable altered was whether the return person/disk was the same or different from that seen previously. Stimulus presentation order was consistent for each infant but randomized between infants. Individual demonstrators were randomly assigned to conditions but remained consistent across days within each infant. Each infant saw one person who always produced LPS, and one person who always produced TP, except in 3.5% of sessions in which the model was not available and therefore the modeling was carried out by a different person. This occurred in 1 test session for 10 infants, 2 sessions for 7 infants, 3 sessions for 1 infant, and 4 sessions for 1 infant (out of 12 sessions total). Excluding these sessions or infants did not significantly alter the results. Similar to the social conditions, in the nonsocial condition one colored disk always served as the familiar disk, and one always served as the unfamiliar disk. Models were counter-balanced in their roles (familiar/stimulus-period or unfamiliar/return-period) across subjects. All sessions were videotaped using a Sony Digital Video camcorder (either a ZR600 or HDR-CX560V). Videos were focused in on infants' faces only; stimuli were not visible in videos to allow for blind coding.

### Coding Reliability

We coded gestures off-line, frame-by-frame (30 frames per second) from video, using the Noldus Observer XT; Leesburg, VA. Coders scored all occurrences of infants' gesturing (LPS, TP). LPS was operationally defined as a high frequency opening and closing of the mouth in which the lips parted and rejoined within 2 sec. TP was operationally defined as a clear forward thrust of the tongue in which the tongue protruded beyond the lips. Observers had at least 6 months of previous experience with macaque infants and were familiar with their gestures. Observers were blind to the stimulus (condition or return person/disk type). Inter-observer reliability was assessed for 20% of infants (*n* = 18 total). The average observer agreement for gesture frequencies was high for LPS (*r* = .936, *p*<.001, *n* = 196) and TP (*r* = .951, *p*<.001, *n* = 196). For analysis, we averaged across all test days and adjusted to a common time frame (gesture rate per 20 sec).

### Data analysis

Rather than classifying infants as imitators and non-imitators, as in previous papers [(e.g., 4, 16]), we analyzed the data using a different approach, which did not presuppose an a priori differentiation of infants into gross, macroscopic categories (imitators and non-imitators). The following analyses took into account the possibility that infants' imitative responses could be expressed within a behavioral continuum. We computed two imitative indices (i.e., imitation strength scores), one for LPS and one for TP, using the average gesture rates across days, with the following formulas: 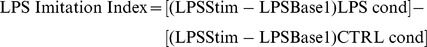


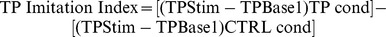


For LPS Imitation Index, we first calculated a difference score: LPS produced in Stimulus and subtracted from it LPS produced in Baseline1. This difference score was computed for the LPS and CTRL conditions, and we subtracted the CTRL condition from the LPS condition to obtain the difference of the difference scores. The resulting value is positive if there was a greater imitative response in the LPS condition, and negative if there was a greater imitative response in the CTRL condition. We calculated the TP Imitation Index in the same way: TP gestures produced in the Stimulus period and subtracted from it TP produced in Baseline1, and subtracted this difference score in the CTRL condition from this difference score in the TP condition.

We scored infants' visual tracking of the social partner (or disk); we labeled individual test sessions as tracking if the infant watched the demonstrator or disk depart at the beginning of the break period, which had to include infants turning their heads by ≥90° in the correct direction. We only examined sessions in which the return person was unfamiliar, as it was in this condition that human infants previously demonstrated differential gesturing as a function of tracking [10]: human infants who tracked the first model's exit may have been more aware that the social partner had changed than infants who failed to track the original model. All t tests are two-tailed, unless otherwise indicated.

## Results

We first examined whether infants gestured more overall, or produced more LPS gestures specifically, during the return period when the return person was the same, compared to when the return person was different. A 2×3 mixed-design analysis of variance (ANOVA) on overall gesture rates (both LPS and TP combined) during the return period, with the between subjects factor of Return person type (Familiar, Novel) and the within-subjects factor of Condition (LPS, TP, CTRL) revealed no main effects or interactions, *p*s>.10. A 2×3 mixed-design ANOVA on LPS rate during the return period with the between subjects factor of Return person type and the within-subjects factor of Condition revealed no main effects or interactions, *p*s>.10. A 2×2 mixed-design ANOVA on the rate of matching gestures (LPS produced in LPS condition and TP produced in TP condition), with the between subjects factor of Return person type and the within-subjects factor of Condition (LPS, TP) revealed no main effects or interactions, *p*s>.10. Together, these results suggest that overall, infants are not more likely to initiate an interaction with a familiar social partner than with a novel social partner, they do not preferentially perform matching gestures they previously saw, and they do not respond with increased LPS.

We next examined infants in the condition in which a different person returned (*n* = 36) to test our hypothesis that infants' tracking of the first model's exit was predictive of the infants' “probing” gesturing (i.e., producing previously modeled gestures) directed at the novel return person. Specifically, we predicted that in sessions in which infants tracked the first model's exit—and therefore infants were more aware that the social partner may have changed—infants would produce fewer of the previously modeled gestures, compared to sessions in which infants did not track the first model's exit. All but one infant exhibited tracking behaviors in at least one (but never in all) test session. In the LPS condition, the model was tracked 50.6% of the time, in the TP condition the model was tracked 47.1% of the time, and in the CTRL condition the model was tracked 40.0% of the time. A 3×2×2 mixed design ANOVA (excluding the one infant who did not track), with factors Gesture (LPS, TP), Condition (LPS, TP, CTRL), and Model tracking (Tracked, Not-tracked) revealed no main effect or interactions with Model tracking, *p*s>.10.

**Figure 2:**
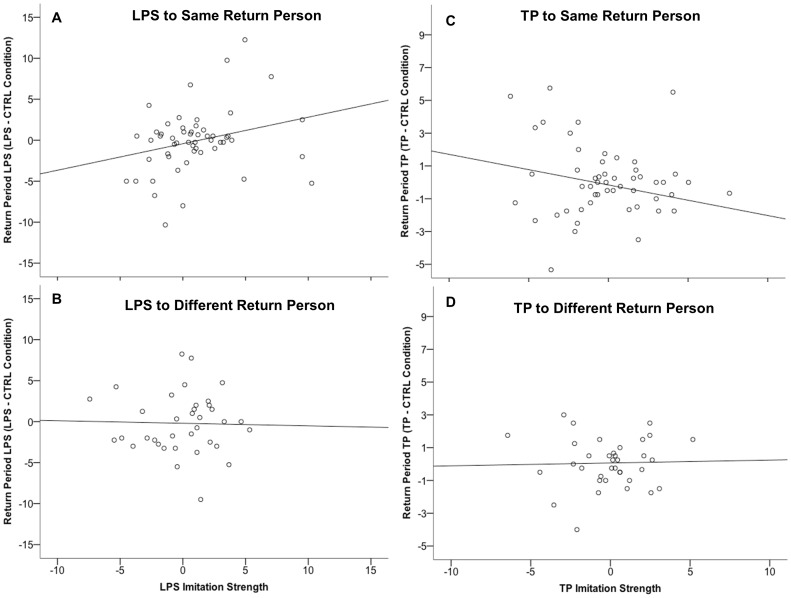
Scatterplots of the relationship between imitation strength and gesture rates during the Return period. Imitation strength is reported separately for lipsmacking (LPS; plots A and B) and for tongue protrusion (TP; plots C and D), and was calculated using the LPS and TP Imitation Index (see main text). Higher scores indicate stronger imitation. Only for LPS was there a stronger correlation when the return person was the same (A) compared to when the return person was different (B), *p*<.001; for TP there was no difference in the correlation coefficients when the return person was the same (C) or different (D), *p* = .17.

Finally, we carried out a series of linear regressions to examine the association between imitation strength and facial gesture production during the still face return period. When the return person was the same there was a modest positive correlation between the strength of LPS imitation (i.e., the LPS Imitation Index) and the rate of LPS during the return period (LPS in Return period for LPS condition minus CTRL condition), *r*(52) = .267, *p* = .025, one-tailed, Figure 2A, but was not significant when the return person was different, *r*(34) = −.025, *p* = .44, one-tailed, Figure 2B. That is, in the LPS condition, when the return person was the same (*n* = 54), approximately 7.1% of the variability in LPS during the return period was predicted by the strength of LPS imitation. The LPS correlation coefficient when the return person was the same was significantly greater than the correlation coefficient when the return person was different, *p*<.001. There was also a correlation between the strength of TP imitation (i.e., the TP Imitation Index) and the rate of TP during the return period (TP in Return period for TP condition minus CTRL condition), when the return person was the same, *r*(52) = −.229, *p* = .048, one-tailed, Figure 2C, and no correlation when the return person was different, *r*(34) = .028, *p* = .44, one-tailed, Figure 2D. However, the TP correlation coefficient when the return person was the same did not differ from the correlation coefficient when the return person was different, *p* = .171. This result suggests that the strength of LPS imitation, but not TP imitation, may be associated with interest in initiating interactions with a familiar social partner.

## Discussion

Our results show that overall, social partner identity did not affect rhesus macaque infants' gesture rates, matching gesture rates, or specifically LPS gesture rates after a brief delay, suggesting that rhesus macaques in the first week of life do not appear to use imitation for the purpose of identifying social partners. For human infants, tracking a social partner's location has been associated with social partner recognition and the production of ‘probing’ gestures upon a new partner's return [8], [10]. In the present study we found that macaque infants' tracking of the model's exit was not predictive of gesture matching during the return period; thus, our results do not support the hypothesis that macaque infants use neonatal imitation to identify social partners. However, in previous work with human infants the return person produced a new facial gesture [10], whereas in the present study the return person produced a still face. It is, therefore, difficult to directly compare the results of these studies. An additional limitation of the present study is that return person type was a between-subjects factor, which limited our statistical power.

Nonetheless, we found evidence that both imitation abilities and social partner recognition may be related, as they may both reflect more general social interest. We found that infants' LPS imitation skill positively correlates with LPS displays produced in response to the still face of the same social partner, but not when the social partner changed. Though this correlation was only moderate in size, our results reveal that one-week-old macaque infants' LPS imitation skill appear to be related to their to ability to remember, after a delay, not only that LPS actions produced [4], but also the individual who produced the actions, namely, that a person returning is the same person with whom they previously interacted.

Thus, although we do not believe that infant macaques generally use imitation for partner identification purposes, some macaque infants can distinguish the identities of social partners, likely using visual cues. Instead, though it remains to be tested, we propose that an infant's neonatal imitation after a delay period may be the consequence of the rewarding nature of the previous interactions that, by inducing positive affect in the infant, stimulate the infant to solicit the same partner in turn-taking behaviors. Infants may learn to address behaviors to individuals who are willing to engage in positive interactions. Neonatal imitation may therefore help to maintain social interactions between infants and caregivers, promoting social bonding [5], [6]. It is possible that because LPS is a natural and affiliative gesture for macaques [14], they may be more sensitive to this gesture, compared to TP, and this may reflect a stronger expression of affiliation towards the more familiar social partner relative to a new social partner. Though admittedly speculative, it is possible that neonatal imitation reflects important individual differences—perhaps differences of the mirror neuron system—which are predictive of a more general developmental pattern of interpersonal skills. Indeed, in recent work we found LPS imitators, compared to non-imitators, were more visually attentive to social and nonsocial stimuli [15]. Future work, employing other measures of social partner discrimination, such as visual paired comparison face discrimination tests, would bring additional insights into the relationship between imitative skill and social identity discrimination.
